# Effect of Strength and Endurance Training Sequence on Endurance Performance

**DOI:** 10.3390/sports12080226

**Published:** 2024-08-20

**Authors:** Vidar Vikestad, Terje Dalen

**Affiliations:** Department of Physical Education and Sport Science, Faculty of Teacher Education and Arts, Nord University, 7600 Levanger, Norway; vidar.vikestad@student.nord.no

**Keywords:** endurance performance, concurrent training sequence, endurance training, strength training, VO2max

## Abstract

This review investigates the effect of two different concurrent training sequences on endurance performance. The sequences investigated are Endurance–Resistance (ER) and Resistance–Endurance (RE). A literature search is conducted of the SPORTDiscus and Medline databases. The included studies are randomized control trials, which compare the effect of ER and RE on at least one endurance performance variable. A PEDro scale is used to assess the methodological quality of the articles in this review. Of a total of 152 articles identified during the initial screening, 15 studies meet the inclusion criteria. These studies include 426 participants (298 males and 128 females), with 212 of the participants training with ER and 214 with RE. The results are presented as the percentage change of the mean from pre- to post-test. All the studies show an improvement in endurance from pre to post for both interventions, except for the RE group in one study. This review finds small and non-conclusive sequence effects between ER and RE, suggesting that the sequence of concurrent training is not of great importance in relation to endurance performance.

## 1. Introduction

Endurance athletes are constantly seeking more effective ways to improve their performance. Different approaches and methods can lead to enhancement, and different approaches affect athletes differently across various sports. When athletes and coaches are seeking further improvements in performance, a natural approach would be to make some adjustments to the training. For endurance athletes, this often means adjustment of the intensity, frequency, and volume [1]. However, another approach that has gained attention is the addition of strength training to the training routine.

Studies have, over the last decade, highlighted the importance of implementing strength training in the training routine of endurance athletes to optimize their endurance performance [2,3,4,5,6,7,8]. The potential benefits of strength training in terms of endurance performance have been attributed to several factors, including improved exercise economy, anaerobic capacity, lactate threshold, maximal strength and speed, rate of force development, and increased time to exhaustion, while not showing any negative outcomes [6,9]. Improvements in endurance performance from strength training also seem to be similar for both males and females [7]. This suggests that endurance athletes should try to implement strength training into their weekly training routine to optimize their endurance performance.

Studies have found interfering effects of concurrent strength and endurance training, suggesting that the concurrent training approach, compared to performing resistance training alone, could mitigate the gains in muscle mass, strength, and power [10,11]. The dose of endurance training is an important factor in relation to the interfering effect on strength training adaptations, as hypertrophy, power, and strength have been shown to be impaired by higher doses of endurance training more than lower doses [11]. Power is reported to be a major variable affected by concurrent strength and endurance training [11,12]. Furthermore, it is suggested that the velocity of the contraction during maximal effort might be an important factor concerning the magnitude of the interference [12]. The interfering effect, which can inhibit adaptation from the strength training, seems to be specific to the body parts used during the endurance training [11]. This was also confirmed in the meta-analysis by Sabag et al. [13], stating that incorporating high-intensity endurance training into the strength training routine did not negatively impact the upper body strength and hypertrophy, although negative effects were found mainly in the lower body.

From an endurance performance perspective, it is important to note that strength and hypertrophy gains do not necessarily contribute to enhanced performance for endurance athletes when incorporating strength training into their routine. In weight-bearing endurance sports where the power-to-weight ratio significantly impacts performance, body mass is an important factor, and athletes can often be afraid to gain extra body mass due to fearing that it will negatively affect their performance [14]. Therefore, the addition of extra body mass caused by hypertrophy from strength training could have a negative effect on endurance performance. This concern was highlighted by Rønnestad and Mujika [6], who stated that the addition of strength training to an endurance training regime does not seem to cause a significant increase in total body mass, even though small increases in muscle hypertrophy in the main target muscles are often observed.

Several previous studies have demonstrated reduced strength adaptations from performing endurance training concurrently with strength training, compared to strength training alone [15,16]. The homeostatic or metabolic and neuromuscular effects of endurance training might affect physiological responses and thereby also adaptations [17]. In general, it has been suggested that one should aim to separate the endurance from the strength training sessions optimally on different days to minimize the interfering effects, but, if possible, it is recommended to place the strength and endurance sessions with at least 6 h of recovery between them [18]. The sequence of same-session concurrent strength and endurance training have also proven to be an influencing factor concerning the training adaptations gained from concurrent strength and endurance training [15,16].

While strength training over time is likely to improve endurance performance and exercise economy [6], there is evidence that the sequence of endurance and resistance training acutely affects the determinants of endurance performance [19,20]. For example, it has been shown that even with six hours between strength and endurance exercise of a different order in running (strength–running and running–strength), resistance training six hours before endurance training impaired both the running economy and muscle force generation capacity [21]. When endurance exercise is performed after strength training without adequate resting time between workouts, the local muscle damage can reduce the running economy and consequently performance [20,22]. For many endurance athletes, limited time for training can be a challenge when planning their program. Full-time professional endurance athletes might not have the opportunity to dedicate a whole day to just strength training due to the high demand for a certain volume of endurance training, while amateur athletes often have work and other commitments that limit their time window for training, which forces them to train with little to no rest between the endurance and strength sessions. Considering the nuanced interplay between time constraints, performance outcomes, and sequencing in concurrent training, this review offers valuable insights for athletes, coaches, and researchers alike. By exploring the impact of the sequence of concurrent endurance and strength training, specifically comparing the effects of strength training followed by endurance training versus the reverse order, this review contributes practical knowledge to inform more effective and feasible training program designs. This brings up an important question regarding the planning of strength training in an endurance training routine. The aim of this review is to examine the impact of the sequence of concurrent endurance and strength training, specifically comparing the effects of strength training followed by endurance training versus endurance training followed by strength training, on endurance performance.

## 2. Materials and Methods

This review used the PRISMA (Preferred Reporting Items for Systematic Reviews and Meta-Analyses) 2020 updated guidelines as a framework to improve the transparency and reporting of the study [23]. While most of the checklist items were followed, relevant components that were appropriate for the research design, data sources, and analysis were included.

### 2.1. Literature Search

In order to answer the research question, a literature search was performed using the SPORTDiscus and Medline databases on 29 February 2024. No starting year was set for the literature search. The search terms employed were as follows: (Endurance exercise OR endurance training OR aerobic exercise OR aerobic training) AND (Strength exercise OR strength training OR resistance exercise OR resistance training) AND (Sequence OR order) AND (Concurrent OR concomitant). Additionally, the search was restricted to peer-reviewed studies published in English. The initial search identified 149 articles. This process involved a single comprehensive search (one-step search) using all the specified search terms simultaneously. The articles then underwent a thorough screening process based on the pre-defined inclusion criteria. The screening process started with the removal of duplicates, and then headlines and abstracts were scanned for relevance. Studies that did not seem relevant based on the headline and abstract were excluded, while the studies that still seemed to be relevant went on to further analysis. The final step consisted of a full-text review of the article for the eligibility criteria, as shown in Table 1. Furthermore, three additional studies were identified through citation searching from the reference lists of other studies and included in the analysis after undergoing the same screening process.

### 2.2. Inclusion and Exclusion Criteria

For this study, only training intervention studies were included, so no studies investigating acute effects were considered. To ensure an adequate timeframe for endurance and strength adaptations, a minimum intervention duration of eight weeks was established as an inclusion criterion. The selected studies were required to incorporate a concurrent training protocol performed sequentially. Specifically, studies had to include both an endurance training–resistance training (ER) sequence and a resistance training–endurance training (RE) sequence. There were no restrictions set regarding participants’ age or fitness level, as most studies were conducted on untrained participants. Studies that included participants with any mentioned diagnosis, illness or injury were excluded from the review. The studies had to include the pre-test and post-test for at least one endurance performance parameter, or an endurance performance test.

### 2.3. Review Process

**Figure 1:**
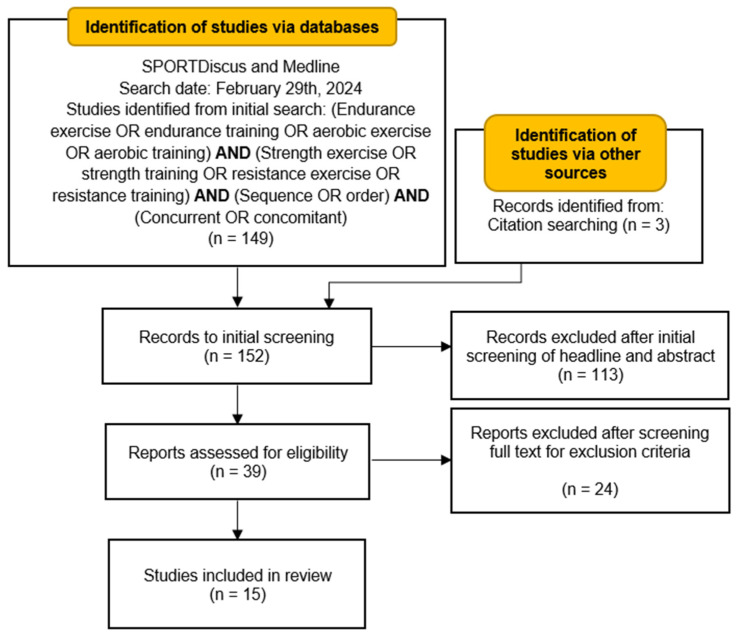
PRISMA flowchart describing the search, screening, and exclusion process for the studies included in this review.

The screening process for this review was conducted by one of the authors, who assessed the identified studies based on the predefined eligibility criteria. All the studies identified from the initial search were first screened by reading the headline and skimming through the abstract. The second screening was performed by reviewing the full text of the selected studies for eligibility (see Figure 1).

### 2.4. Quality Assessment

To assess the methodological quality of the articles in this review, a modified version of the Physiotherapy Evidence Database (PEDro) scale [24] was used (see Table 2), where the criteria regarding blinding were excluded since they were deemed irrelevant for the methodological quality of the included studies. The criteria of the PEDro scale were reviewed for each article to determine whether they were satisfied. A points system was used for marking the criteria. A “+” indicated that the criterion was clearly stated and satisfied, a “÷” indicated that the criterion was not satisfied, and a “?” indicated uncertainty regarding whether the criterion was satisfied. Only studies that scored a minimum of 5 points on the PEDro scale were included in this review, ensuring a baseline level of methodological quality.

### 2.5. Data Extraction

The lead author conducted the extraction and compilation of data from the included studies, converting the information into two comprehensive spreadsheet tables. The extracted data covered aspects like the weekly frequency of strength and endurance sessions, the duration of the transfer time between sessions, and specifics of the training protocols for both the strength and endurance sessions, as elaborated in Table 3. 

Additionally, each study’s data encompassed details such as the number of participants, sex distribution, age demographics, training status descriptions, intervention duration, and results of endurance tests for both the ER and RE groups. A detailed breakdown of this information is provided in Table 4. 

### 2.6. Participants

The 15 included studies involved a total of 426 participants, with 212 following the ER protocol and 214 the RE. Out of the 426 participants, there were 298 males and 128 females. The age and training status varied a lot across participants, but no studies were conducted on elite or professional athletes. From the descriptions in the studies, they were described as everything from untrained to physically active, with no mentioned underlying health issues.

### 2.7. Statistical Analyses

This systematic review refrains from conducting a meta-analysis or statistical analyses due to the significant variations in the endurance tests performed across the included studies and the heterogeneity in the presentation of data. Results from the individual studies are reported in Table 4 as the percentage change of the mean values from pre- to post-test. The level of significance, denoted by asterisks and a section symbol, indicates significant differences: * = 0.05 (pre-post), ** = 0.01 (pre-post), and § = 0.05 (between-group), marking studies with significant pre-post or between-group differences when mentioned in the studies.

## 3. Results

### 3.1. Study Characteristics

The studies included in this systematic review showed variation in their intervention protocols and participant characteristics. In terms of the participant demographics, the studies included both male and female subjects with varying age ranges. In five of the studies, the participants are described as young or students, with an age span ranging from teenagers and participants in their early 20s in some of the studies, to participants with an average age of over 60 years in other studies. The training status varied across the studies, encompassing healthy individuals, sports students, moderately healthy individuals, and untrained participants. The specific exercise protocols were detailed, outlining the warm-up and cool-down durations, intensity progressions, and specific exercises for both the endurance and strength training components. The studies exhibited differences in their use of outcome measures. The endurance outcomes of the included studies are presented in Table 4, which also provides details regarding the number of participants, their age, sex distribution, duration of the interventions and training status of the participants.

### 3.2. Maximal Oxygen Uptake

As one of the most commonly used endurance performance parameters, the oxygen uptake was tested the most in the included studies, as it was measured in ten of the fifteen studies included in this review, either in the form of VO2max, or VO2peak. All the studies showed a significant increase from pre to post in both the ER and RE groups, except the RE group in the study by Ruiz-Alias et al. [33]. The ER group in that study showed a significant increase in VO2peak pre to post, while the RE group did not, but did not differ significantly from the RE. Two studies showed significant between-group effects on the oxygen uptake. One of those studies found a significant difference favoring ER over RE [28], while the other one favored RE over ER [25]. The study by Chtara et al. [28] also found a significant between-group effect for the velocity at maximal oxygen consumption (vVO2max), favoring ER over RE, with both groups showing a significant increase from pre- to post-test.

### 3.3. Other Endurance Measures

One of the studies [28] tested the time trial performance and found a significant performance increase from pre to post in both the ER and RE groups. The magnitude of the change was larger in the ER group, which resulted in a significant between-group effect favoring ER over RE. In six of the studies, either the maximal aerobic power (MAP) or maximal power on incremental test (Wmax) was tested. All the studies showed significant improvement from pre to post for both the ER and RE groups. One study [32] found a significant between-group effect for the morning-ER group over the morning-RE group from pre to post. The mean increase in the evening-ER group was significantly larger than the evening-RE group from week 13–24, but not from pre to post. Four studies tested the time to exhaustion (TTE). All of these showed significant improvements from pre- to post- test. The study by Küüsmaa et al. [32] was the only one of these studies to show a significant between-group effect favoring ER over RE, both for the morning and the evening groups. Two studies tested the lactate threshold (LT) and second ventilatory threshold (VT2). Both showed a significant increase from pre to post for both the ER and RE groups, but no differences between the two groups.

## 4. Discussion

The aim of this review is to examine the impact of the sequence of concurrent endurance and strength training, specifically comparing the effects of strength training followed by endurance training versus endurance training followed by strength training, on endurance performance.

No significant differences between the ER and RE groups were found in any of the measured outcomes in 11 of the 15 studies. One study [25] favored RE over ER and showed a significant between-group effect for VO2max. However, this was the only study and measurement that favored RE over ER. On the other hand, three studies found significant between-group effects favoring ER over RE for the following outcomes: Wmax [32], TTE [31], and VO2max, vVO2max and TT performance [28].

One similarity between the studies that favored ER is that the endurance training was performed at high intensity, as described in Table 2. Several explanations can be proposed for why these studies, with these characteristics, showed a larger sequence effect. It is plausible that a higher-intensity workout, compared to a lower-intensity one, is more challenging to complete, and therefore, fatigue buildup from an initial strength training session might negatively affect the high-intensity session to a larger degree. The strength training prior to the endurance session might have affected the performance in the endurance session negatively, which we have reason to assume it would, based on studies showing impaired endurance performance after strength exercise [21,40]. Therefore, it is important to consider the potential impact of initial fatigue from previous strength training on subsequent endurance sessions, especially those at maximal exertion. However, none of these studies specified the intensity the two groups (ER and RE) performed their workouts at. Thus, it cannot be inferred that this was the reason for the findings in this review, and further research is needed to explore whether the sequence of concurrent training might affect performance due to initial fatigue and to what extent this might influence endurance training outcomes.

In contrast, the study by Banitalebi et al. [25], which favored RE, was conducted with low training intensities for both the endurance and strength sessions, and the sessions lasted only 16–30 min. A potential weakness with that study is that the ER and RE groups do not appear to be similar in terms of the VO2max at baseline, as shown in Table 1, with ER performing better than RE. This difference in aerobic fitness between the groups might have made the initial gains from the endurance training easier to gain for the RE group, which started from a lower level. Another important aspect of this study is that investigating the effect of the ER vs. RE sequence on endurance performance was not the main goal of the study. Several included studies primarily focused on other measurements to address their research questions, which is potentially suboptimal for studying the sequence effect on endurance performance.

Another area where the study favoring RE differs from those studying ER is the duration of the intervention. While the study favoring RE [25] lasted over eight weeks, the studies that favored ER lasted for 24 [31,34] and 12 weeks [28], respectively. The participants were also much younger in the studies favoring ER, as described in Table 3. And for the study by Chtara et al. [28], the participants had a higher baseline VO2max, at 51 mL/kg/min for both the ER and RE groups, compared to 29.07 ± 1.88 (ER) and 24.60 ± 1.35 (RE) in the study by Banitalebi et al. [25]. The other two studies that favored ER did not measure the oxygen uptake, but from the description of the participants (Table 3), it is reasonable to assume that they also had better baseline fitness.

The reviewed studies often investigated other research questions than what sequence of concurrent training is best for improving endurance performance. So, more studies of better quality, and studies investigating the concurrent training sequence effect on performance as their main research question, are needed to gain more knowledge on this topic. The research questions often concerned health outcomes or other hormonal measurements, often among elderly, overweight, or untrained participants. In many of the studies, the strength outcomes were a lot more thoroughly tested, with more tests and measurements than performed for the endurance outcomes. One or more endurance measures were taken but were usually not the main focus area of the studies. The endurance measurement most commonly used by the included studies in this review was an oxygen uptake test, which is considered an important performance parameter and a measure of cardiorespiratory fitness [41]. It is considered a good predictor of performance in some endurance sports like uphill running races [42]; however, it might not be a good performance predictor in, for example, longer lasting events like mass-start bike races [43].

This review only found a small sequence effect between ER and RE, only for when the endurance training was performed at a high intensity, and otherwise no difference between the concurrent training sequences. Despite this, it is important to mention that for strength adaptations, the concurrent training sequence can play a more important role. Studies have shown that RE can have a significant favorable effect over ER on strength adaptations [12,44,45]. This can be due to hormonal responses such as reduced mTOR signaling due to AMPK induced by endurance training [46]. Hypertrophy gains typically take considerable time to develop, which can be even more challenging for endurance athletes experiencing the interference effect from their endurance training [10,11]. Knowing this, even endurance athletes could consider performing strength training prior to the endurance session if they must do both consecutively, if gaining muscle mass is of interest to the athlete. As previously mentioned, an increase in locomotive muscle mass has been thought to be a positive adaptation from strength training for endurance athletes [7]. Therefore, endurance athletes who are seeking to increase their muscle mass in the locomotive muscles in their main sport could consider performing strength training prior to endurance exercise. This might also be worth considering in training early in the preparation period and further from competitions when strength adaptation might be more of a priority.

The sequence of concurrent training within the same session seems to have a negligible impact on endurance and a small effect on strength parameters. On the other hand, separating the sessions by at least 6 h has been shown to be beneficial for minimizing the acute interference effect, which can negatively affect such parameters as running economy [18,20,21]. Strength training, which induces muscle damage, can reduce performance and oxygen uptake even 48 h after exercise, depending on the degree of muscle damage caused by the strength training [40]. One can therefore argue for the importance of an adequate resting period after the strength training to perform the endurance session with optimal quality, particularly those of high intensity.

A potential concern when studying untrained, older and/or overweight participants is that factors such as daily activities, a general warm up or self-transportation to and from the training location may affect the findings due to their similarity in duration and intensity to the intervention, and therefore, they might give similar responses to the intervention. Given the low training doses for both the strength and endurance interventions, it can be argued that a short general warm up before the strength training, which was performed in several of the studies, made it hard to investigate the sequence effect of ER and RE. For example, a walk from a bus stop or a parking lot, which might include some longer stairs and a long hallway toward the training facility, can suddenly mean that just getting to the location of the workout itself might look quite similar in terms of the intensity and duration as the intervention. In the study by Banitalebi et al. [25], the participants trained at 40% of 1 RM for the strength training, and for 16 min at 45% of VO2max for the endurance part, during the initial weeks of their intervention. This corresponds to an endurance intervention intensity of 13.1 and 11.7 mL/kg/min for ER and RE group, respectively, which is an intensity similar to a typical walking pace [47,48]. This makes it very likely that when studies investigate the sequence effect of strength and endurance training in untrained/overweight individuals, especially with as small training doses as used in some of the included studies, daily activity might give some similar responses as the intervention if the intervention is set at a low enough duration and intensity. This poses difficulties in examining any sequence effect, particularly when daily non-training activities are performed in close proximity to the training intervention.

Too low a training intensity and volume might contribute to another problem when trying to investigate the sequence effect. According to the study by Coffey and Hawley [49], the adaptations resulting from single-mode training (e.g., strength or endurance alone) and concurrent training are largely comparable in the first days/weeks of training for an untrained individual. They further hypothesize that it could be because the body does not differentiate between strength and endurance-like adaptational signals, likely because strength and endurance training is conducted at quite a moderate volume and intensity. This might partially explain some of the findings of this review. For example, the lack of any sequence effect might be somewhat due to the fact that a lot of the studies were performed with a low-volume and low-intensity intervention and conducted on untrained participants. For the studies that found a sequence effect favoring ER, high-intensity endurance training was a common denominator of all three studies. Those studies were also among the studies that stretched over the longest duration, which is a possible benefit, especially if the participants are untrained.

This review has some potential limitations. Firstly, the evidence provided is scarce, with only 15 studies meeting the eligibility criteria. In this review, 9 of the 15 included studies only used one endurance measurement, and they used various tests, so there were limited studies investigating the same outcome. The participants, duration of interventions and intervention protocols were also widely different across the studies, which makes it more difficult to make comparisons between them. Another limitation is that the effect sizes of the between-group effects could not be derived from all the studies, so data had to be presented as the change of the mean pre- to post-tests and level of significance of the between-group effect. Because of the substantial heterogeneity of the methods used across the studies, no statistical test for a meta-analysis could be conducted, and the findings were just interpretations of the results made by the authors. Furthermore, only one of the authors conducted the screening and PEDro assessment, which could introduce potential bias and limit the reliability of the selection and quality assessment process.

The concurrent training sequence effect appears to be marginal, with small potential gains or losses to be made from choosing one sequence over the other. Since the participants in the studies included in this review were mainly untrained, they often experienced large improvements in the endurance parameters from pre to post. This might make it harder to observe a small sequence effect between the two groups. A preferable study design to investigate the sequence effect could have been one that was conducted on elite or professional athletes, where one would not expect to see a large increase in the pre to post values due to the prescribed training, and therefore, one might have been able to investigate the sequence effect of the concurrent training a bit more effectively.

The main objective of this review was to examine the sequence effect of ER compared to the RE training sequence. In general, this review finds small and non-conclusive sequence effects between ER and RE, suggesting that the sequence of concurrent training is not of great importance in relation to endurance performance. The findings of this review, which primarily included untrained to moderately trained individuals, show largely small to no effect from one sequence over the other. However, the concurrent training sequence might be a relevant concern for both professional and amateur athletes when planning their training schedule. It is important to note that none of the studies in this review were conducted on elite or high-level athletes, and the training volume used for the interventions would have been very low for most athletes in most situations. This limitation makes it challenging to extract relevant data to provide recommendations for endurance athletes. Further research on this topic, particularly involving participants of higher fitness levels, is needed to better understand the impact of a concurrent training sequence for endurance athletes at a higher level.

If, for practical reasons, both training modes must be performed with little to no rest between them, one could argue that it is advisable to prioritize the highest priority workout first, and preferably to perform strength training the same day as a low-intensity and low total-load endurance workout. This approach might help minimize the interference between the two modes of exercise. In cases where strength training must be performed close to a high-intensity endurance session, it may be preferable to perform the endurance session first, if endurance performance is a higher priority for the athlete than strength adaptations. One alternative can be to perform the strength and endurance sessions using mainly different muscle groups to minimize the interference. For example, cycling for the endurance part followed by upper-body strength training, or hand cycling followed by lower-body strength training.

## 5. Conclusions

This review finds small and non-conclusive sequence effects between ER and RE, suggesting that the sequence of concurrent training is not of great importance in terms of endurance performance. This review also highlights the need for more and better research on the effect of concurrent training sequence on endurance performance. As of now, there is not enough evidence to draw any conclusions.

## Figures and Tables

**Table 1:** Participants, interventions, comparisons, outcomes, study (PICOS) design table describing the inclusion and exclusion criteria.

PICOS	Inclusion Criteria
Participants	Participants of any age or fitness level. Exclusion of participants with any diagnosis, illness, or injury.
Interventions	Training intervention studies with a minimum duration of eight weeks, comparing concurrent endurance and strength training sequence.
Comparisons	Studies comparing a strength–endurance sequence (e.g., resistance training followed by endurance training) with an endurance–strength sequence (e.g., endurance training followed by resistance training).
Outcomes	Studies must investigate at least one endurance performance parameter or an endurance performance test.
Study design	Training intervention studies of at least eight weeks, comparing ER and RE sequences. No age or fitness restrictions. Excluded studies with diagnosed illnesses or injuries and those investigating acute effects.

**Table 2:** The modified PEDro scale used for the quality assessment. “+”, criterion satisfied; “÷”, criterion not satisfied; “?”, unclear whether criterion is satisfied. The criteria regarding blinding were dimed irrelevant due to being n/a (not applicable). The arrow “↓” in the total score row indicates the criteria listed below.

Banitalebi et al. (2016) [25]	C Lee et al. (2020) [26]	Cadore et al. (2012) [27]	Chtara et al. (2005) [28]	Davitt et al. (2014) [29]	Eklund et al. (2016) [30]	Küüsmaa et al. (2016) [31]	Küüsmaa-Schildt et al. (2017) [32]	Ruiz-Alias et al. (2022) [33]	Schumann et al. (2014) [34]	Schumann et al. (2015) [35]	Tarasi et al. (2011) [36]	Wilhelm et al. (2014) [37]	Salamat. (2017) [38]	Esazadeh et al. (2020) [39]	
6	6	8	6	6	6	5	6	6	5	5	6	5	6	6	Total score, Criterion↓
+	+	+	+	+	+	+	+	+	+	+	+	+	+	+	Eligibility criteria were specified
+	?	+	÷	+	?	÷	÷	?	?	?	+	?	+	?	Subjects were randomly allocated to groups
÷	÷	÷	÷	÷	÷	÷	÷	÷	÷	÷	÷	÷	÷	÷	Allocation was concealed
?	+	+	+	+	+	+	+	+	+	+	+	+	+	+	Groups were similar at baseline
n/a															Blinding of subjects
n/a															Blinding of therapists
n/a															Blinding of assessors
+	+	+	+	÷	+	÷	+	+	÷	÷	+	÷	?	+	Key outcome measured for ≥85% of the subjects
+	+	+	+	+	+	+	+	+	+	+	+	+	+	+	Treatment allocated as intended
+	+	+	+	+	+	+	+	+	+	+	+	+	+	+	Between-group measure
+	+	+	+	+	+	+	+	+	+	+	÷	+	+	+	Point and variability measure

**Table 3:** Summary of the training interventions used in the included studies.

Study	ER/Week	RE/Week	Endurance Training Mode/Strength Training	ER Protocol	RE Protocol	Sequence/Transfer
Banitalebi et al. (2016) [25]	3	3	Ergometer cycling Whole-body strength training	10 min warm up and cool down. 16 min at 45% of VO2max, progressing to 30 min by the end of the training protocol.	10 min warm up and cool down, 50 min exercise. Bench press, leg press, bent over lateral pulldown, bilateral biceps curl and bilateral triceps push down. Performed at 40% of 1 RM the first week, progressing to 75% of 1 RM during the eight weeks.	2 min between sessions
C Lee et al. (2020) [26]	3	3	Ergometer cycling Whole-body strength training	Warm up: 5 min at 75 watts. HIIT: 8–13 × 2 min at 85–97% of VO2peak. 1 min rest between intervals.	Warm up: 5 reps at~75% of training load. Main part: 3–4 sets of 12–6 repetitions, with 2 min rest. All sets were max reps for given load. Exercises session one: Leg press, bench press, seated row, leg extension, leg curl Exercises session two: Leg press, dumbbell chest press, lat. pulldown, lunges, leg curl	3 h between sessions
Cadore et al. (2012) [27]	3	3	Ergometer cycling Whole-body strength training	Ergometer cycling. First two weeks: 20 min at 80% of HRVT2, progressing to 25 min at 85–90% in week 5–6, and 30 min at 90% on week 7–10, and in week 11–12 40 min at 100%.	Bench press, inclined leg-press, seated row, knee extension, inverse fly, leg curl, triceps curl, biceps curl and abdominal exercises. Week 1–2: 2 sets of 18–20 RM, week 3–4: 2 sets of 15–17 RM, week 5–7: 2 sets of 12–14 RM, week 8–10: 3 sets of 8–10 RM, week 11–12: 3 sets of 6–8 RM. 1.5–2 min recovery between sets	Same session
Chtara et al. (2005) [28]	2	2	Running Whole-body strength training	Interval: five times 50% of TTE duration at 100% of vVO2max, with same duration active recovery at 60% of vVO2max.	15–20 min warm up. Exercises: Abdominal strengthening, hip extension, back extensors, half squats, forward alternated arm flexions, forward walking slits. 6 exercises, 30–40 s work/20–30 s rest. 4 series, with 2 min rest between series.	N/A
Davitt et al. (2014) [29]	4	4	N/A Whole-body strength training	30 min of moderate to moderate–high intensity at 70–80% HR reserve.	Chest and back, shoulders and arms, lower body. 3 sets of 8–12 repetitions for 5–6 different exercises using a load equal to 90–100% of 10 RM. 60–90 s rest between sets.	No more than 5 min
Eklund et al. (2016) [30]	2→3	2→3	Ergometer cycling Lower-body strength training	30 min of cycling at 65% of Wmax.	Leg press. Explosive strength: (3 × 10 repetitions at 40% 1 RM with 3 min rest between sets), maximal strength (4–3 × 3 repetitions at 75–90% 1 RM with 3 min rest between sets), Hypertrophy training: (4–3 × 10 repetitions at 75–80% 1 RM with 2 min rest between sets)	5–10 min
Küüsmaa et al. (2016) [31]	2→3	2→3	Ergometer cycling Whole–body strength training	30–50 min sessions. Interval: 4 × 4 min at 85–100% of HRmax with 4 min of active recovery at 70% of HRmax. Continuous training: at 65–80% of HRmax. One extra interval session was added in the last 12 weeks.	3 lower body and 4–5 upper body exercises. Week 1–4: muscular endurance 40–70% 1 RM. Week 5–8: Hypertrophy training 70–85% of 1 RM. Week 9–12: Hypertrophy and maximal strength 75–95% of 1 RM. Week 13–24: Same program with intensity adjusted to match current strength level.	No more than 5–10 min
Küüsmaa-Schildt et al. (2017) [32]	2→3	2→3	Ergometer cycling Whole-body strength training	30–50 min sessions. Interval: 4 × 4 min at 85–100% of HRmax with 4 min of active recovery at 70% of HRmax. Continuous training: at 65–80% of HRmax. One extra interval session was added in the last 12 weeks.	3 lower body and 4–5 upper body exercises. Week 1–4: muscular endurance 2–3 sets of 10–20 reps at 40–70% 1 RM. Week 5–8: Hypertrophy training 3–4 sets of 10–15 reps at 70–85% of 1 RM. Week 9–12: Hypertrophy and maximal strength 3–5 sets of 3–8 reps at 75–95% of 1 RM. Week 13–24: Same program with intensity adjusted to match current strength level.	No more than 5–10 min
Ruiz-Alias et al. (2022) [33]	3	3	Running Whole-body strength training	Sprint interval training. Week 1–2: 4 × 30 sec all out, 4 min active recovery. Week 3–4: 5 × 30 sec all out, 4 min active recovery. Week 5–8: 6 × 30 sec all out, 4 min active recovery.	Bench press and back squat. Week 1–2: 4–5 sets at 60% 1 RM, 5–6 RIR Week 3–4: 5–6 sets at 70% 1 RM, 3–4 RIR Week 1–2: 5–6 sets at 80% 1 RM, 2–3 RIR Week 1–2: 6 sets at 80% 1 RM, 1–2 RIR 2 min rest between sets	10 min
Schumann et al. (2014) [34]	2→3	2→3	Ergometer cycling Whole-body strength training	Week 1–7 steady state < 65–67% HRmax. Week 8–24: high intensity interval training was added. Session duration: 30–50 min. Habitual physical activity was maintained throughout the study.	Exercises: Leg press, knee extension, vertical press, lat pulldown, and core exercises. Week 1–2: 2–4 sets of 15–20 reps at 40–60% of 1 RM. Remaining weeks: Hypertrophy training 2–5 sets of 8–10 reps at 80–85% of 1 RM, or maximal strength: 2–5 sets of 3–5 reps at 85–95% of 1 RM. 1.5–4 min rest between sets.	No more than 10 min
Schumann et al. (2015) [35]	2→3	2→3	Ergometer cycling Whole-body strength training	Week 1–7 steady state <65–67% HRmax. Week 8–24: high-intensity interval training was added. Session duration: 30–50 min. Habitual physical activity was maintained throughout the study.	Exercises: leg press, knee extension, vertical press, lat pulldown, and core exercises. Week 1–2: 2–4 sets of 15–20 reps at 40–60% of 1 RM. Remaining weeks: Hypertrophy training 2–5 sets of 8–10 reps at 80–85% of 1 RM, or maximal strength: 2–5 sets of 3–5 reps at 85–95% of 1 RM. 1.5–4 min rest between sets.	No more than 10 min
Tarasi et al. (2011) [36]	N/A	N/A	Running Whole-body strength training	10 min warm up. Duration and intensity progressed from 16–30 min and 65–80% of HRmax. 10 min cool down.	10 min warm up. Exercises: leg press, chest press, half squats, sit-ups. 2–3 sets per exercise, intensity progressing from 50% 1 RM to 80% 1 RM, with reps decreasing from 10–6. 60–90 s rest between sets. 10 min cool down.	N/A
Wilhelm et al. (2014) [37]	2	2	Ergometer cycling Whole-body strength training	Week 1–3: 20 min at 85% HRVT2. Week 4–6: 30 min at 85% HRVT2. Week 7–9: 30 min at 95% HRVT2. Week 10–12: 40 min at 95% HRVT2.	Exercises: lat pulldown, bench press, elbow extension, bicep curls, leg press, knee extension, and knee flexion. Week 1–3: 2 × 15–18 RM. Week 4–6: 2 × 12–15 RM. Week 7–9: 3 × 10–12 RM. Week 10–12: 3 × 8–10 RM. 1–2 min rest between sets.	5 min
Salamat et al. (2017) [38]	3	3	Treadmill running. Whole-body strength training	55% of HRmax for 25 min progressing to 85% for 45 min at the end of the protocol.	Bench press, biceps and triceps flexion–extension with weights, underhand cable pulldown, leg press and core exercises, which were performed with 50 to 80% of 1-RM, increasing intensity with 10% every 2 weeks	10 min active rest
Esazadeh et al. 2020 [39]	3	3	N/A Whole-body strength training	15 min warm up First sessions were 20 min at 65% HRmax, then progress to 40 min at 80% by the end of the training protocol	15 min warm-up, 45 min exercise. Exercises: biceps curl, triceps pushdown, lat pulldown, lateral raise, incline chest press, leg extension, leg curl and calf raise. 3–5 min rest between sets.	10 min

The table contains: ER/week, RE/week, training modes, ER protocol, RE protocol, and sequence/transfer. Abbreviations: ER (endurance training), RE (resistance training), VO2max (maximum oxygen consumption), VO2peak (peak oxygen consumption), HR (heart rate), RM (repetition maximum), HIIT (high-intensity interval training), vVO2max (velocity at maximum oxygen consumption), RIR (reps in reserve), Wmax (maximal power on incremental test), TTE (time to exhaustion), 1 RM (one-repetition maximum), vVO2peak (velocity at peak oxygen consumption), RPE (rating of perceived exertion), N/A (not available), HR (heart rate), HRmax (maximal heart rate), HRVT2 (heart rate at second ventilatory threshold).

**Table 4:** Description of the participants, duration of the intervention, and results of each included study.

Study	ER N=	RE N=	Mean Age	Training Status and Description	Duration Weeks	ER vs. RE	Results (% Change of Mean Average)
Banitalebi et al. (2016) [25]	9 ♀	10 ♀	60.34	Healthy	8	ER < RE	VO2max: ER ↑17% ** RE ↑29.3% **§
C Lee et al. (2020) [26]	10 ♂	10 ♂	24.5 ±4.7	Moderately healthy	8	ER = RE	VO2peak: ER ↑11% *, RE ↑10% * MAP: ER ↑22.3% *, RE ↑18.7% * LT: ER ↑15.7% *, RE ↑15.1% *
Cadore et al. (2012) [27]	13 ♂	13 ♂	64.7 ±4.1	Healthy	12	ER = RE	VO2peak: ER ↑8.3% **, RE ↑7.7% ** Wmax: ER ↑24.1% **, RE ↑18.9% **
Chtara et al. (2005) [28]	10 ♂	10 ♂	21.4 ±1.3	Sports students	12	ER > RE	VO2max: ER ↑13.6% **§, RE ↑10.7% ** TT (time): ER ↓8.6% **§, RE ↓ 4.6% * TTE: ER ↑33.4% **, RE ↑25.9% ** vVO2max: ER↑10.4% **§, RE ↑8.2% **
Davitt et al. (2014) [29]	13 ♀	10 ♀	19.8	Inactive college students	8	ER = RE	VO2max: ER ↑15.8% * RE ↑15.5% *
Eklund et al. (2016) [30]	15 ♀	14 ♀	18–40	Untrained	24	ER = RE	Wmax: ER ↑ 21% ** RE ↑16% **
Küüsmaa et al. (2016) [31]	21 ♂	21 ♂	Young men	Untrained	24	ER > RE	TTE: mER ↑19.7% ** (§ vs. eRE), mRE: ↑19.4% ** eER ↑26.9% **§, eRE ↑18.2% **
Küüsmaa-Schildt et al. (2017) [32]	20 ♂	21 ♂	32.3 ±5.6	Physically active, healthy	24	ER > RE	Wmax: mER ↑21.6% **§, mRE ↑14.9% ** eER ↑20.5% **, eRE ↑15.5% ** (eER: § from eRE week 13–24)
Ruiz-Alias et al. (2022) [33]	4♂ + 5♀	8♂ + 3♀	21	Healthy young adults	8	ER = RE	VO2peak ER ↑9.1% ** RE ↑3.3%
Schumann et al. (2014) [34]	16 ♂	18 ♂	30 ± 5	Healthy	24	ER = RE	VO2max: ER ↑6.1% *, RE ↑6.4% ** TTE: ER ↑9% **, RE ↑10% ** MAP: ER ↑13% **, RE ↑16% **
Schumann et al. (2015) [35]	16♂ + 15♀	18♂ + 13♀	30	Healthy	24	ER = RE	(♂) VO2peak: ER ↑5.7% *, RE ↑6.6% * (♀) VO2peak: ER ↑10.7% *, RE ↑9.2% * (♂) Wmax: ER ↑11.9% *, RE ↑15.9% * (♀) Wmax: ER ↑20% *, RE ↑15.4% *
Tarasi et al. (2011) [36]	10 ♂	10 ♂	17.22 ± 0.94	High school students	8	ER = RE	VO2max: ER ↑3.69% * RE ↑3.75% **
Wilhelm et al. (2014) [37]	11 ♂	12 ♂	65.4	Healthy	12	ER = RE	VO2peak: ER ↑6.9% *, RE ↑8.9% * VT2: ER ↑8.5% *, RE ↑7.2% * TTE: ER ↑9.9% *, RE ↑9.3% *
Salamat et al. (2017) [38]	13 ♂	13 ♂	N/A	Young healthy	8	ER = RE	VO2max: ER ↑21.77% * RE ↑15.64% *
Esazadeh et al. 2020 [39]	11 ♀	10 ♀	N/A	Postmenopausal women	8	ER = RE	6 min walk test: ER ↑11.87% * RE ↑7.73% *

Abbreviations: ♀ (female), ♂ (male). Within group effects: * (*p* = 0.05), ** (*p* = 0.01). Between-group effects: § (*p* = 0.05), ↑ (increase), ↓ (decrease), > (favoring ER over RE), < (favoring RE over ER), = (no difference between-groups), ER (endurance before resistance training), RE (resistance before endurance training) mER/RE (morning ER/RE group), eER/RE (evening ER/RE group), VO2max (maximal oxygen uptake), VO2peak (peak oxygen consumption), vVO2max (velocity at maximal oxygen consumption), Wmax (maximal power on incremental test), MAP (maximal aerobic power), LT (lactate threshold), TT (time trial), TTE (time to exhaustion), VT2 (second ventilatory threshold).
